# Quality of Research Practice – An interdisciplinary face validity evaluation of a quality model

**DOI:** 10.1371/journal.pone.0211636

**Published:** 2019-02-01

**Authors:** Pär Mårtensson, Uno Fors, Emelie Fröberg, Udo Zander, Gunnar H. Nilsson

**Affiliations:** 1 Department of Entrepreneurship, Innovation and Technology, Stockholm School of Economics, Stockholm, Sweden; 2 Department of Computer and Systems Sciences, Stockholm University, Stockholm, Sweden; 3 Department of Law, Languages and Economic Statistics, Stockholm School of Economics, Stockholm, Sweden; 4 Department of Marketing and Strategy, Stockholm School of Economics, Stockholm, Sweden; 5 Department of Neurobiology, Care Sciences and Society, Karolinska Institutet, Stockholm, Sweden; Universidad Veracruzana, MEXICO

## Abstract

There are few acknowledged multidisciplinary quality standards for research practice and evaluation. This study evaluates the face validity of a recently developed comprehensive quality model that includes 32 defined concepts based on four main areas (credible, contributory, communicable, and conforming) describing indicators of research practice quality. Responses from 42 senior researchers working within 18 different departments at three major universities showed that the research quality model was–overall–valid. The vast majority believed all concepts in the model to be important, and did not indicate the need for further development. However, some of the sub-concepts were indicated as being slightly less important. Further, there were significant differences concerning ‘communicable’ between disciplines and academic levels, and for ‘conforming’ between genders. Our study indicates that the research quality model proposes the opportunity to move to a more systematic and multidisciplinary approach to research quality improvement, which has implications for how scientific knowledge is obtained.

## Introduction

### Background

Ultimately, research ought to contribute to the base of scientific knowledge. ‘What is good research?’ is relevant to ask both in academic contexts and practical organizational settings as it relates to, for example, research activities at universities and R&D activities in companies. There is an ongoing discussion of how to evaluate research, including research productivity [1], performance [2] and impact [3], and the effects of such measurements [4–6]. Recent research has also highlighted the need for moving these discussions forward by moving to another level of abstraction [7].

### Dimensions of the quality of research practice

There are several challenging aspects underlying the guiding question of what good research is. Specifically, it is important to identify (first) what exactly research is, (second) good according to whom, and (third) good according to what criteria. Thus, this paper addresses these three underlying aspects, although particularly emphasizes the latter regarding criteria.

The first aspect–*‘what research is’*–is broad, and also opens up the discussion on science versus research. Here, we adopt a terminology where science refers to a broader concept other than research–which is the practice of working in a scientific manner. This means that research is what you practice, and the result of this work is science. The distinction between these concepts has been discussed in more detail elsewhere [8–10]. However, by using a structured approach, ‘research’ can be defined in a concept model—including how other related concepts link [10]. There has also been a discussion about rethinking knowledge production in general [11,12], thus putting the question regarding the essence of research into a larger context.

The second aspect–*‘good according to whom’*–relates to the issue of different stakeholders assessing whether they perceive a piece of research as ‘good’ or not. There are many different stakeholders involved in this: Researchers, executives, funding bodies, policymakers, and journal editors–just to name a few. When evaluating research, a common question regards if there is a theoretical contribution. This in itself opens up for another related question about what precisely a theoretical contribution is, as there are ongoing discussions about what it constitutes and how it should be evaluated [13–15]. Further, when evaluating R&D projects, a parallel question is if there is a practical or financial contribution from the project. Nevertheless, this study does not specifically focus on the different types of contributions, but rather on how to evaluate the research practice.

Recent debates concern the knowledge dissemination processes of the academic profession [16]. Researchers (too) focused on publishing their research in the right journals often generate their research questions through ‘gap-spotting’, that is, trying to find a gap in existing literature to fill [17]. Instead, research questions through problematization can arguably generate more ‘interesting’ questions [18], therefore turning ‘gap-spotters’ into ‘reflexive inventors’ [17]. Editorial boards and reviewers within scientific journals play important roles in securing publications of ‘interesting’ research, and thus need to be composed of expert individuals in order to meet this aim [19]. This second aspect points to the need of finding ways of helping and supporting these experts who evaluate the quality of research, specifically as peer-review processes traditionally serve to evaluate the quality of research [20].

The third aspect–*‘good according to what criteria’–*concerns the criteria different stakeholders apply when evaluating research quality. In the U.K., the Research Excellence Framework (REF) since 2014 assesses research institutions on its outputs (65%), impact (20%), and environment (15%) [21]. These evaluations are summarized in a rating system where each institution receives one to four stars, where four stars means ‘quality that is world-leading in terms of originality, significance and rigour’. In terms of the dimensions discussed above, this means that the third aspect about criteria is defined in a number of dimensions.

Other common dimensions are *rigor* and *relevance*. Although, these are often treated as if they are opposed. Rigorous research refers to research that is conducted with a rigorous scientific approach and thereby trustworthy or credible. Relevant research refers to that which is perceived as relevant in non-scientific contexts, or in ‘practice’, and thus contributory. Over the past several decades, there has been an ongoing discussion about the gap between rigor and relevance, with some researchers arguing that it is not possible to bridge due to the different nature of the two systems of science and practice [22]. However, some efforts have been made, for example, by framing it as a knowledge transfer issue, where bridging media enables knowledge transfer from science to practice [23]. Further, various educational efforts have also been suggested as approaches in this bridging process [24]. There have also been voices raised to reframe this ‘either/or’ discussion [25], such as including sub-concepts such as readability [26].

The discussion on rigor and relevance has, nevertheless, been ongoing for many years [27], and there have been different calls for research that combines rigor and relevance, sometimes referred to as ‘consumable’ research [28]. Although the debate has prevailed, the language has varied [29]. In some areas, such as management research [7], it seems that a pendulum has been swinging between a focus on rigor or relevance. But, there have also been calls for an antithesis on a higher level of abstraction to move beyond the dialectic debate on rigor and relevance [7]. Indeed, it has been argued that certain research methods are more suitable than others when catering for both rigor and relevance, for example, action research [30]. Moreover, in specific subject areas, recent attempts have been made to take the dialog further, such as in information systems research where shifting focus to proof-of-concept, proof-of-value, and proof-of-use research have been suggested to combine rigor and relevance [31]. For some domains, this seems to be a fruitful way forward, but more generally, there are at least two problems. First, the starting-point is still with the core concepts of rigor and relevance. Second, the three ‘proof-of’ concepts suggested may not be applicable to all disciplines. This, however, is not suggested by Nunamaker Jr et al.’s study [31] which indicates some limitations.

### General starting points

A large body of empirical research emphasizes the fragmentation and contextual base in science and knowledge production, which implicitly challenges the application of a research quality model across scientific disciplines and institutions. Various conceptual terms are used, including ‘thought collective’ [32], developed further to ‘thought worlds’ [33], ‘epistemic communities’ [34,35], ‘occupational communities’ [36,37] and ‘communities of knowing’ [38]. The community of practice approach [39,40] has also become increasingly influential for explaining the relationships between science, practice, learning, and innovating. There has also been a discussion of the need for greater inclusion of perspectives from low and middle income countries in the debate about the essence of research and how it can be distinguished from science and knowledge production [41].Taken together, it is clear to see that there is pluralism in academic thought over similar phenomena.

With the preceding discussion in mind, it is our intention here to carry out an empirical investigation of what unites and divides views on research quality between different disciplines and institutions in terms of evaluation. We therefore aim to obtain some first indications of the degree of fragmentation on the views of research quality between different scientific disciplines and institutions of higher learning by conducting an initial examination of the wider applicability of a research quality model. We argue that guiding principles apply generically to scientific endeavors. Does this mean that economics is the same as cell biology? Or that the medical model can be imported wholesale into the study of business administration? No. It only means that the contention is that all science shares a set of underlying principles of inquiry, while the ways these norms are instantiated vary in clear and important ways. Of course, each field has features that influence what questions are asked, how research is designed and carried out, and how it is interpreted and generalized. Thus, there are powerful contextual factors in the research process. Scholars working in a particular area collectively–as a community–establish the scientific traditions and standards regarding how to apply the guiding principles of what constitutes quality to the particular area of study. The characteristics of the profession affect the nature of the work as well as the conceptions of community. For example, the presence of numerous disciplinary perspectives (e.g., anthropology, psychology, sociology, economics, and neuroscience) means that there are many research frameworks seen as legitimate methods and norms of inquiry. The aim is, therefore, not to debate the relative merits of methods as no method is good, bad, scientific, or unscientific in itself. Rather, it is the appropriate application of method to a particular problem that enables judgments about scientific quality. It is the possibility of arriving at generally applicable models for quality assessment that is in focus in this paper.

### Preceding study

**Fig 1:**
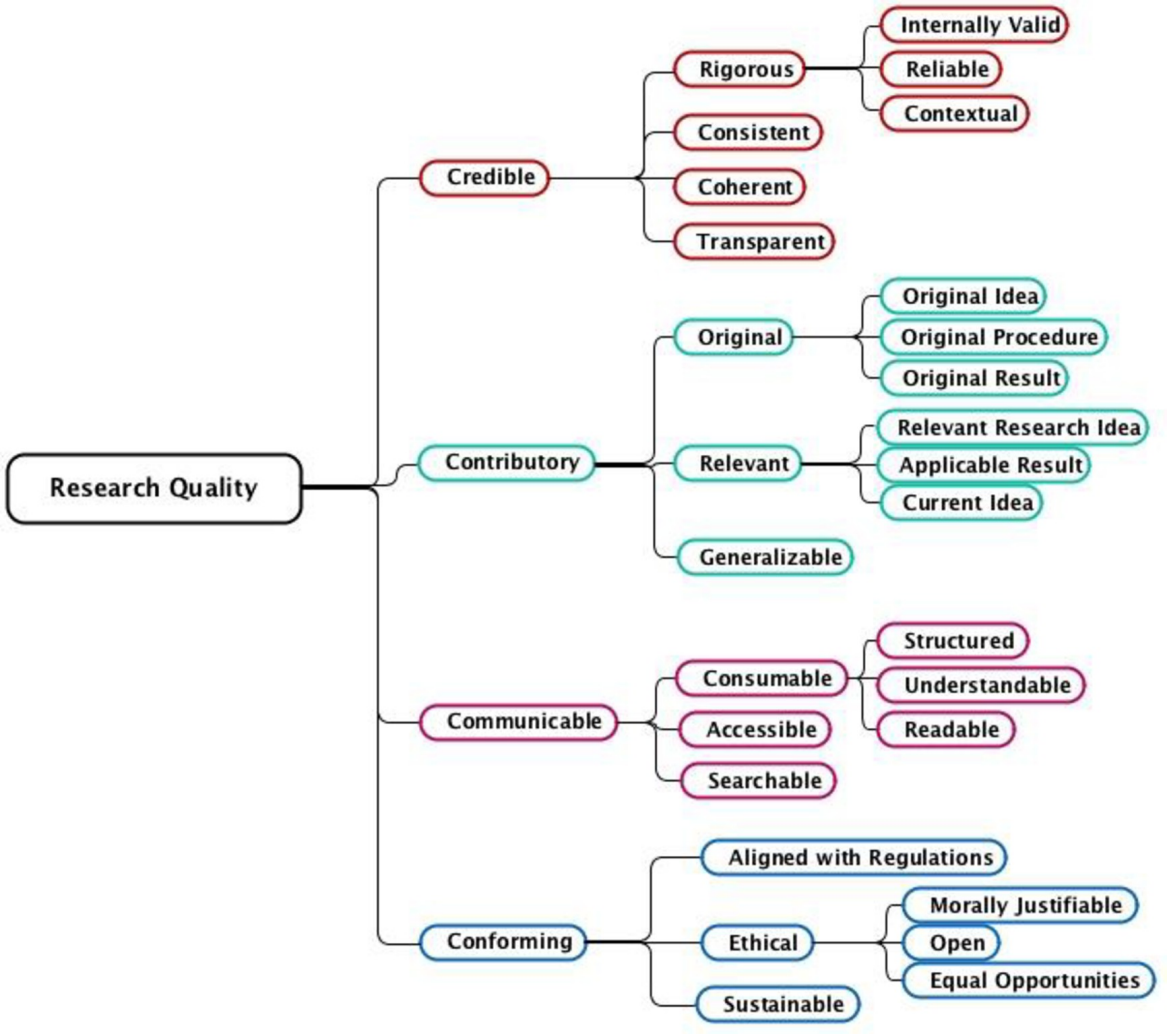
Concept hierarchy of research quality (from Mårtensson et al, 2016).

Mårtensson et al. [10] developed a research quality model including different aspects for the quality of research practice. This research quality model proposes a broad characterization of what constitutes quality in research. Four main areas were distilled, describing research practice in a multidisciplinary context: credible, contributory, communicable, and conforming (Fig 1). The model has the potential of capturing multidisciplinary complexity and dimensions of rigor and relevance, but whether it subjectively portrays what it sets out to measure is yet to be tested. This study therefore proposes to evaluate the face validity of the suggested model by testing how the different dimensions of research quality are perceived among senior researchers in various research fields.

### Context, criteria, and relative weights

There is thus a need for a universal concept model for the quality of research practice including *weights*, *validated criteria*, and *multidisciplinarity*. When assessing the quality of research practice there is the need to be more explicit about the criteria used, not least in light of concerns raised about novice experts in evaluating research [16]. Moreover, there is the need to give stakeholders assessing research quality a better toolbox to assess various aspects of research. And, although some countries have formed national commissions for evaluation, these are often focused on bibliometric analyses to measure research quality [42]. The Research Excellence Framework in the U.K. is an example of a national system with the aim to capture several different dimensions [21]. Nevertheless, there can be huge variations between different countries, as well as between different disciplines and various institutions, illustrated in a case from Poland [43]. In R&D projects, pure research less frequently accomplished initially defined goals than applied research and experimental development did. Technical sciences applied project management methodologies, specifically adaptive methods, more often than all other fields of science [43]. Essentially, with a better toolbox, stakeholders could assess research quality in more detailed ways and not only in a single and simplified dimension between rigor and relevance. That is, the context needs to be taken into account and not one singular dimension can capture this complexity [29].

### Aims

The first aim of this study is to evaluate the face validity of the 32 concepts of a previously published concept model for the quality of research practice among a broader group of researchers. The second aim is to capture if other concepts are lacking in the concept model, and to potentially develop the model with more concepts. For example, the possible need of further development, as well as discipline, gender, and academic level differences were assessed. The third aim is to identify if there are concepts in the current version of the model that a broader group of researchers perceives as not important and could thus be removed.

## Method

Regarding the above-mentioned aims, the following method was adopted.

### Methodological considerations

A questionnaire was developed (see S1 Appendix) that focused on evaluating the 32 concepts of Mårtensson et al.’s [10] model, including a background and open questions that could be added to or removed from the model. The participants evaluated the concepts one by one, rather than the tree structure of the original model, to avoid biases in the concepts’ evaluation. As the study aims to evaluate face validity and develop the concept model further, a broader group of senior researchers from different disciplines were the focus. That is, we did not aim to capture a representative sample of researchers, but rather to make the initial effort of developing the model further by assessing whether it subjectively covered ‘research quality’ according to a somewhat larger audience. We thus used convenience sampling [44] to recruit participants. Specifically, we invited researchers representing different subject areas from different universities. However, all invited participants were limited to senior researchers (i.e., associate professors, full professors, department heads etc.).

This study follows the Swedish law about ethics approval of research about humans (lag (2003:460) om etikprövning av forskning som avser människor) and the ethical guidelines (CODEX) from the Swedish Research Council (Vetenskapsrådet). Namely, researchers should only seek ethics approval when dealing with personal sensitive data or criminal records (3 §), or when intervening physically or mentally on alive, or dead, human beings (4 §). Further, participants were informed about the research and its purpose, approved to participate (all participants were adults), were kept confidential and responses were only used for research purposes.

### Participants

Senior researchers at three leading Swedish Universities within medicine, economics, and the social sciences were invited to participate in the study, totaling 62 invitations. Of them, 42 responded (corresponding to a response rate of 68%). The participants worked at 18 different departments with a broad representation of various academic disciplines: clinical neuroscience; cognitive neuroscience; dentistry; family medicine; epidemiology; health informatics; history; internal medicine; international economic studies; law; management and organization; marketing and strategy; medical education; medical epidemiology and biostatistics; occupational science; psychology; public health sciences; social work; sociology; special education; statistics; and sustainable markets. Table 1 shows the descriptive statistics of the participants’ age, gender, and seniority divided into the three participating universities: Stockholm School of Economics (SSE), Karolinska Institutet (KI), and Stockholm University (SU). It should be noted that the majority of participants from SSE and KI conducted research in business administration (95%) and medicine (88%) respectively, because of the convenience sampling approach.

**Table 1:** Descriptive statistics of participants.

	SSE	KI	SU	Total
**Age (*M*)**	49	57	54	52
**Females (%)**	37	43	44	40
**Professors (%)**	53	86	44	55
***N***	*16*	*19*	*7*	*42*

### Measurements

The questionnaire was constructed with three main parts (the complete questionnaire can be found in S1 Appendix). First, it included background questions related to academic degree, position, research field, age, and gender. Second, it asked the participants to rank 32 concepts on a scale of (1) not at all important, (2) somewhat important, (3) moderately important, (4) very important, and (5) of crucial importance. Finally, the questionnaire had open-ended questions about the 32 concepts. If the participants felt that anything was lacking, additional thoughts about the potential use of the model for various purposes could be added.

Participants were primed to think about scrutinizing research quality in a PhD thesis when evaluating the 32 concepts, as we asked (see Q15): *‘To evaluate the quality of a dissertation in your field*, *how important are the following concepts*?*’* The reason for this was to create comparability as every discipline and subject area has a dissertation (or similar) as the first major research product. Dissertations are, in that sense, general in contrast to academic papers or funding proposals that could be influenced more by the specific discipline. We thus reasoned that priming participants to think about dissertations would enable us to evaluate general research quality as the concept model and its 32 concepts aim to capture. However, we also asked about other possible uses of the research quality model (see Q18): *‘In general*, *for what purpose(s) do you think this type of model could be useful*?*’* This was deemed necessary to, for example, evaluate applications for research funding, if dissertations should pass, and the university’s research, as well as to compare research quality and review scientific manuscripts. Moreover, respondents were also asked questions about the concepts they would like to add or remove.

### Data

All survey responses from the 42 participants, including their gender, university and seniority, are openly available in an SND data deposit [45].

## Results

**Fig 2:**
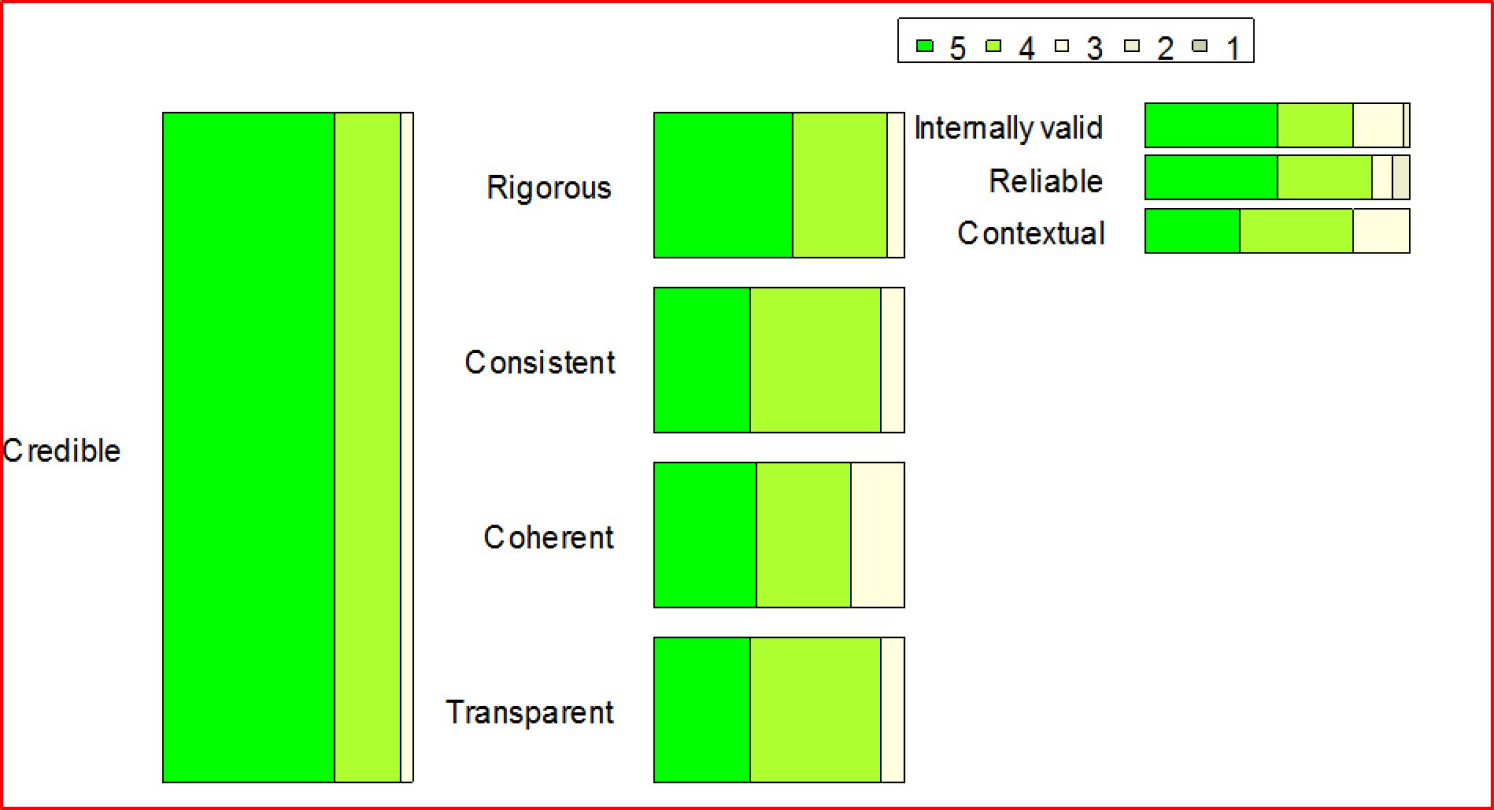
Responses regarding the credible main concept, ranked from 1–5, where 5 is “of crucial importance”.

**Fig 3:**
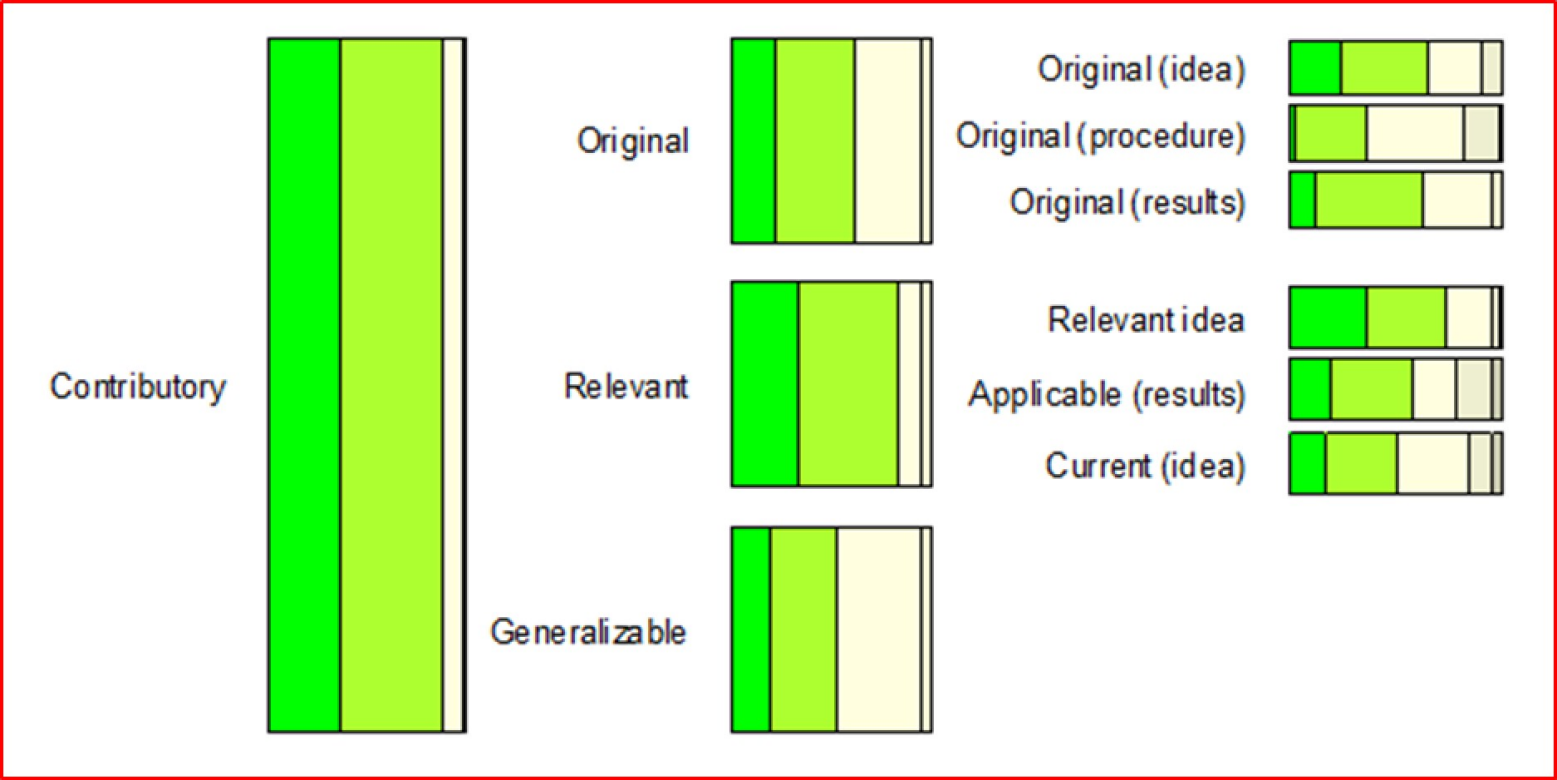
Responses regarding the contributory main concept, ranked from 1–5, where 5 is “of crucial importance”.

**Fig 4:**
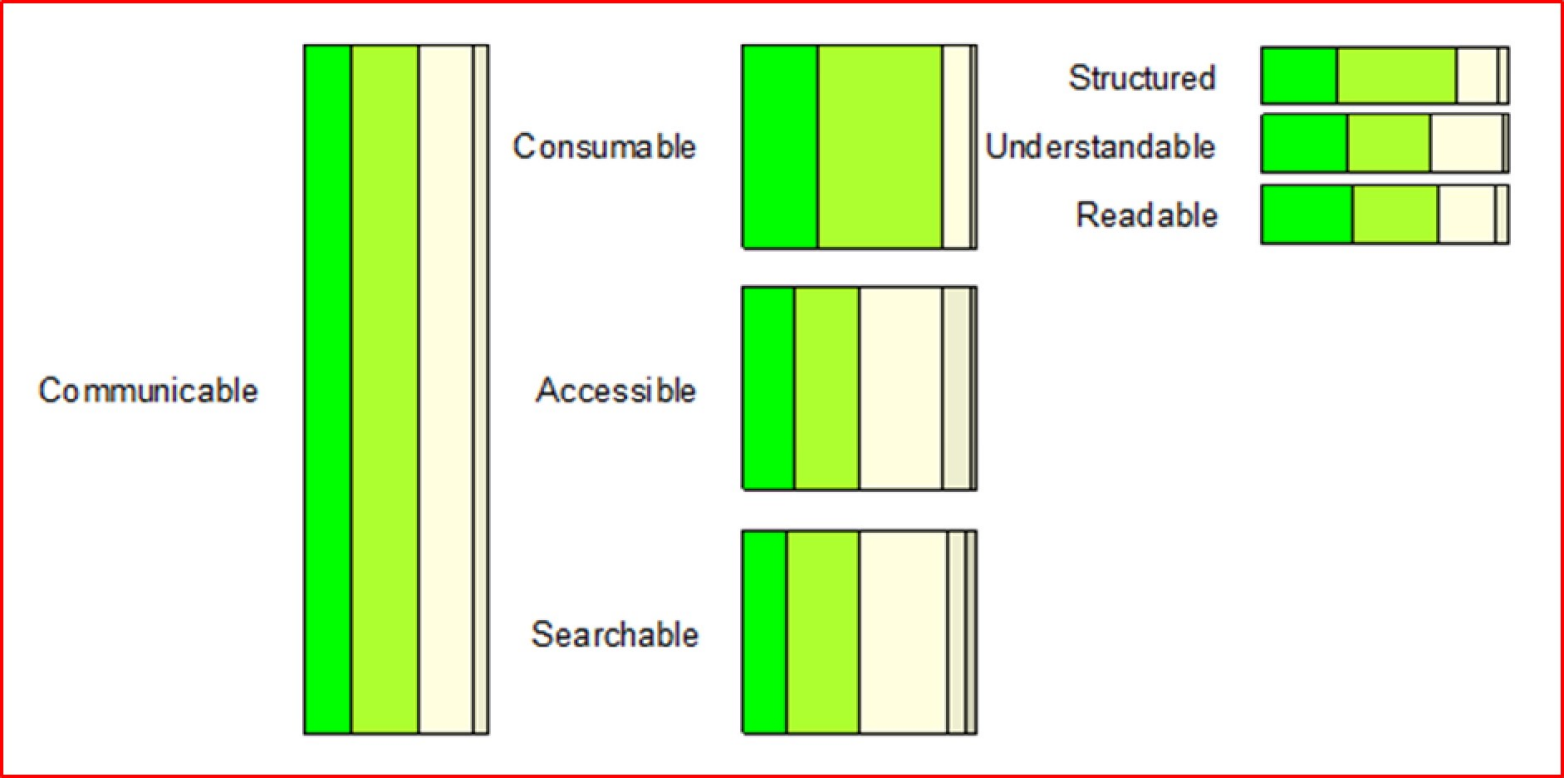
Responses regarding the communicable main concept, ranked from 1–5, where 5 is “of crucial importance”.

**Fig 5:**
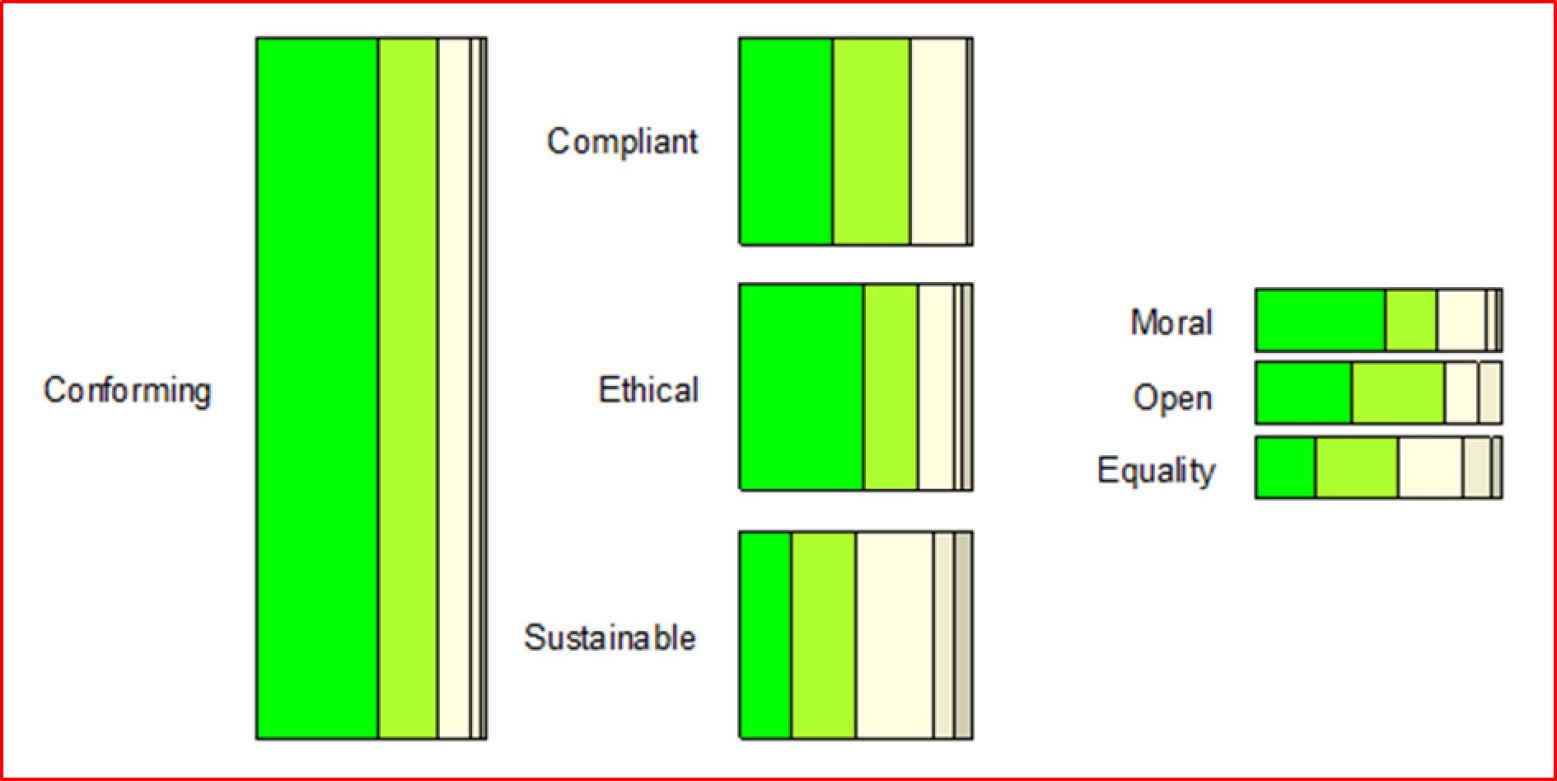
Responses regarding the conforming main concept, ranked from 1–5, where 5 is “of crucial importance”.

Overall, the participants perceived the concept model very positively and the 32 concepts as very important. Given that the tree structure was not revealed to participants in advance, the fact that all 32 concepts were perceived as important is not biased to the four overarching concepts. Over one fifth of the participants indicated all four main concepts as very important or higher (9 out of 42) (please refer to Figs 2–5 and S1 Table for more details). This trend seems to be supported by the high scores for almost all sub-concepts. Exact binomial tests showed that 20 concepts out of all 32 in the quality model were significantly higher ranked than if we had flipped a coin between rankings of crucial importance or very important on the one side, and moderately important, somewhat important, or not at all important on the other. For all exact binomial tests, we have conservatively used a probability of 0.5 for success (i.e., ranking of very important or higher). The sub-concepts with somewhat lower figures, not significantly higher than flipping a coin, are original (including idea, procedure, and results), generalizable, applicable results, current idea, communicable, accessible, searchable, sustainable, and equality. In the results section, we also present binomial tests using a probability of 0.4 for success (that is, all options are equally likely). Further, as a note on Figs 2–5, participants ranked the concepts on a 5-point Likert scale as described, and the responses were reported as 100% stacked bar charts (for exact numbers see S1 Table). The different sizes of the bar charts in the figures have no other meaning than to reflect the concept model of Mårtensson et al. [10].

### Credible

As can be seen in Fig 2, almost all of the 42 participants were very positive to the first main concept of credible as 95.2 percent indicated it to be of crucial importance or very important. Only 4.8 percent indicated it to be moderately important and none indicated it as somewhat important or not at all important. An exact binomial test showed that the proportion of participants that considered credible as very important or higher was significantly greater than those that did not, p < .001. This trend can also be seen in the underlying concepts rigorous, consistent, coherent, and transparent where very few respondents found them to be of moderate importance or less (exact binomial tests showed that the proportions were much higher, p < .001). The only answers indicating any sub-concept to be less than moderately important can be found related to the concepts of internally and reliable, where only one (2.4%) and three participants (7.1%) respectively assigned the sub-concepts to be somewhat important. Still, exact binomial tests showed that the proportion of participants that considered the sub-concepts very important or higher was significantly greater than those who did not, p < .001.

### Contributory

The participants were also very positive to the second main concept of contributory, however not as positive as credible, which can be seen in Fig 3. Almost all participants (88.1%) indicated contributory to be of crucial importance or very important, only 9.5 percent believed it to be of moderate importance, 2.4 percent somewhat important and none indicated it as not at all important. An exact binomial test showed that the proportion of participants that considered contributory as very important or higher was significantly greater than those that did not, p < .001. However, for all sub-concepts–original, relevant, generalizable, original idea, original procedure, original results, relevant research idea, applicable results, and current idea–there were a few participants who indicated these items as somewhat important or not at all important. Exact binomial tests showed that the proportion of participants that considered the sub-concepts as very important or higher was not significantly greater than those who did not, p > .05, for all sub-concepts except relevant (p < .001) and relevant research idea (p < .01). Using a probability of success of 0.4 changes the p-values so that p < .001 for relevant research idea, p < .01 for original (including idea and results) and p < .05 for applicable results. Especially, the sub-concepts original procedure, generalizable, and current idea were seen as less important as 35.7, 52.3, and 50.0 percent respectively assigned them as moderately important, somewhat important, or not at all important.

### Communicable

The third main concept of communicable was also seen as very important or higher by the majority (61.9%) of the participants. However, a two-sided exact binomial test showed that the proportion of participants that considered communicable as very important or higher were not significantly greater than those that did not, p = .1641. However, this result is sensitive to the probability of success. Using a probability of 0.4 changes the p-values so that communicable was significantly higher ranked than expected by chance at 1 percent level, p = .004. As can be seen in Fig 4, there were also positive answers regarding both the main concept and its sub-concepts. Specifically, exact binomial tests showed that the proportion of participants that considered consumable, structured, understandable, and readable as very important or higher were significantly greater than those that did not, p < .001 for consumable and structured, and p < .05 for understandable and readable. Using a probability of success of 0.4 changes the p-values so that p < .001 for understandable and readable. However, for the sub-concepts accessible and searchable, the respondents were less convinced of their importance as only half of the participants indicated these to be of crucial importance or very important.

### Conforming

For the fourth main concept–conforming–there were also very positive responses, and only 21.4 percent of the participants indicated it to be moderately important or less (see Fig 5). An exact binomial test showed that the proportion of participants who considered conforming as very important or more was significantly higher than those that did not, p < .001. The underlying concepts of compliant, ethical, moral, and open were rated high. Exact binomial tests showed that the proportion of participants that rated them as very important or greater was significantly higher than those that did not, p < .001 for ethical and open, and p < .01 for compliant and moral. Using a probability of success of 0.4 changes the p-values so that p < .001 for compliant and moral as well. Interestingly, there were only 50.0 and 57.1 percent respectively, who considered the sub-concepts sustainable and equality of crucial importance or very important.

### Rigor vs. relevance

According to the quality model, rigorous and relevant are sub-concepts of the two main concepts credible and contributory respectively. Credible and rigorous were ranked equally for the majority (78.6%) of participants. Participants ranked credible slightly higher than rigorous, but a Wilcoxon Signed-Rank test did not indicate any statistically significant differences at the 5 percent significance level (two-sided), Z = 1.81, p = .070. Contributory and relevant were ranked equally among over half (52.4%) of the participants. That is, the participants ranked contributory slightly higher than relevant, but a Wilcoxon Signed-Rank test did not indicate any statistically significant differences, Z = 0.59, p = .559.

Table 2 shows the distribution of ranks across credible and contributory. As can be seen, the results indicate that these are both very important concepts in evaluating research quality. There is no evidence that credible and contributory are complementary or in opposition, as the percentage of participants that ranked credible as very important or higher did not differ from the percentage that ranked contributory as very important or higher, χ^2^ (1, n = 42) = 0.28, *p* > .05. Further, one must recall that there were no statistical differences in the ranks between credible and rigorous as well as for contributory and relevant.

**Table 2:** Credible and contributory in a 2-way frequency table (percentages).

	Credible		
Contributory	Moderately important	Very important	Of crucial importance
Slightly important			2.4
Moderately important		4.8	4.8
Very important		21.4	31.0
Of crucial importance	4.8		31.0

On a final note, among the participants that perceived credible as very important or greater, the percentage of participants (27 out of 40, or 67.5%) that perceived original procedure–a sub-concept to contributory–as moderately important or lower was significantly higher than the percentage that perceived it to be very important or greater (13 out of 40 or 32.5%), χ^2^ (1, N = 40) = 4.9, p < .05. In other words, credible was significantly higher ranked than original procedure.

### Comparison between subgroups

**Fig 6:**
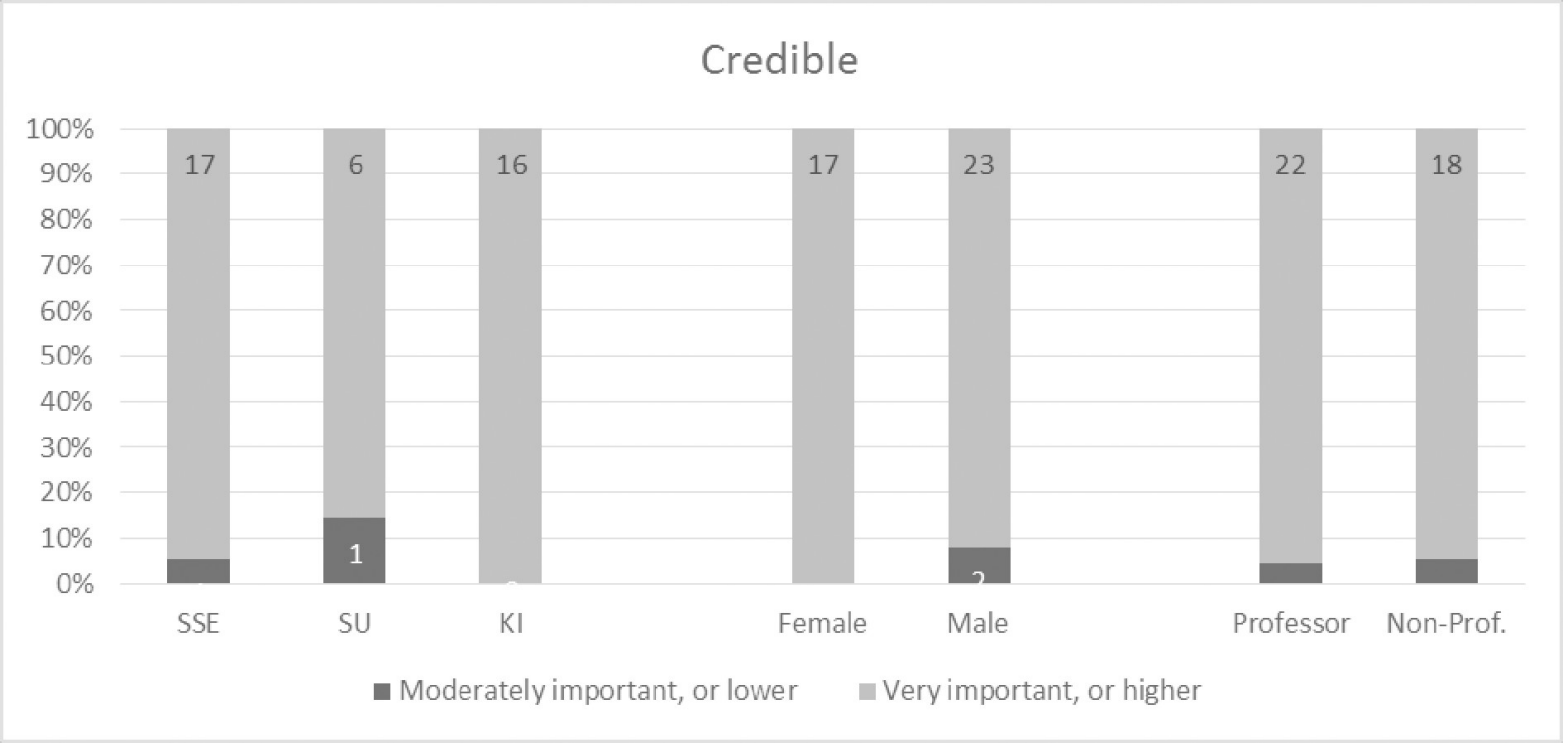
Response rates for credible between universities, gender, and academic level.

**Fig 7:**
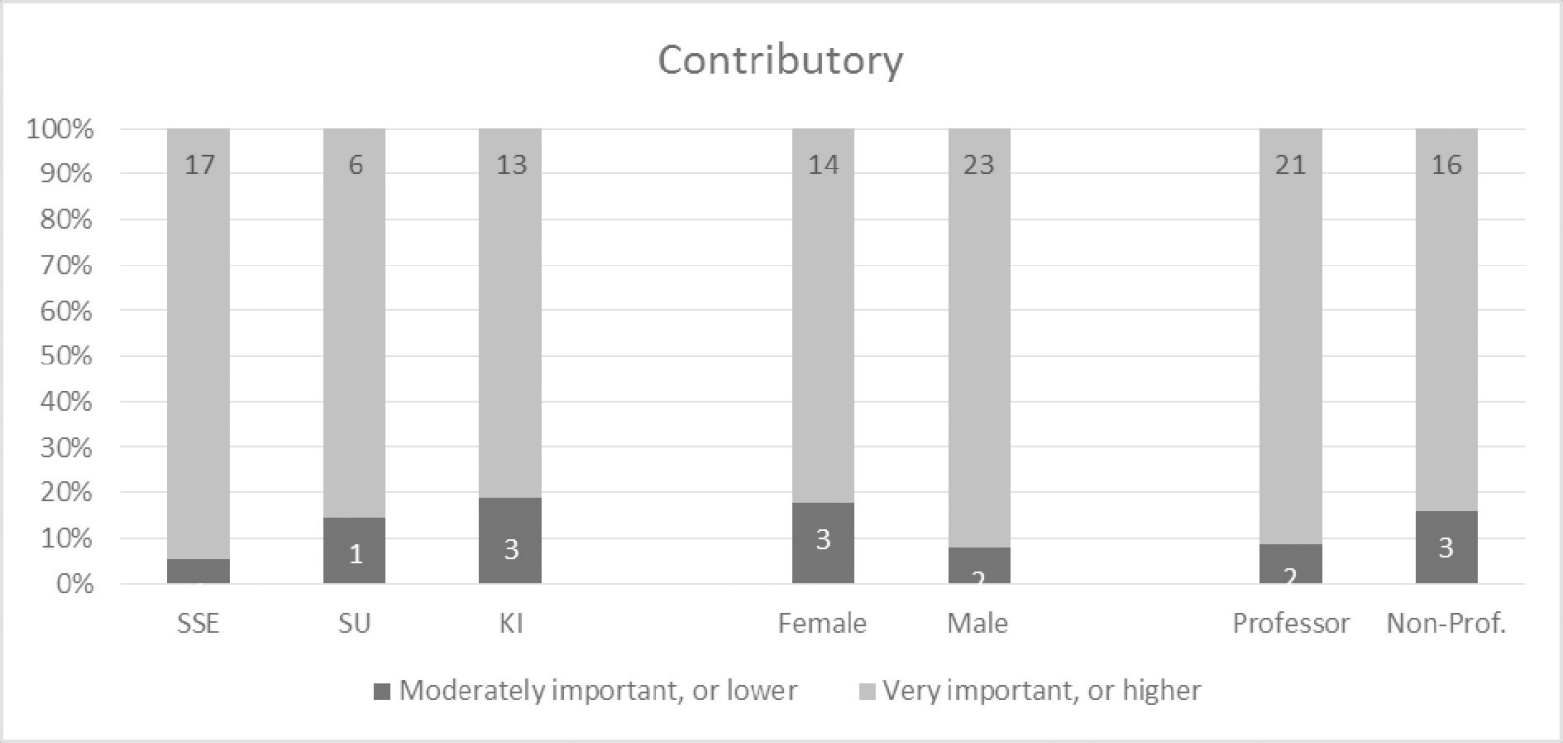
Response rates for contributory between universities, gender, and academic level.

**Fig 8:**
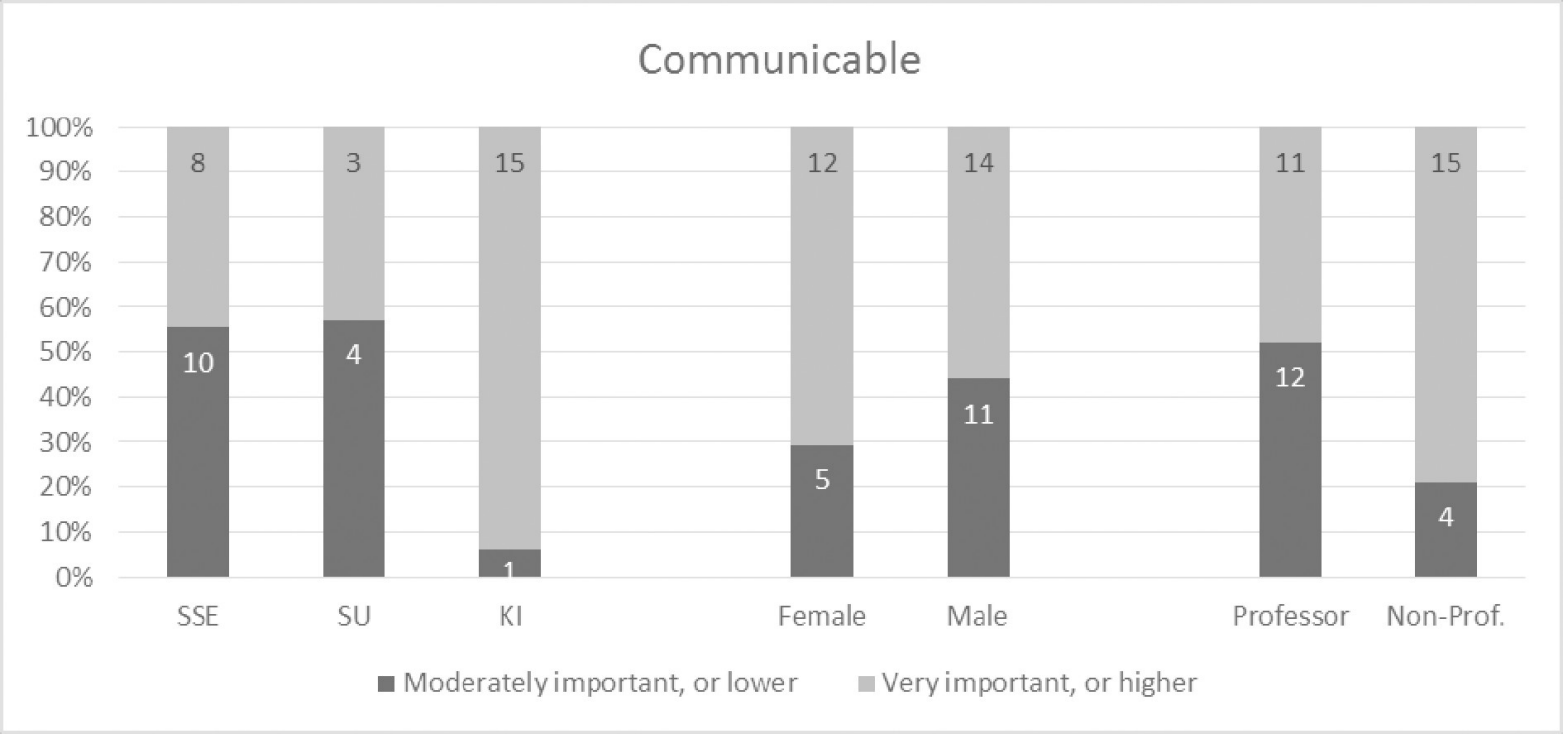
Response rates for communicable between universities, gender, and academic level.

The percentage of participants that considered credible and contributory as very important or greater was generally above 80 percent (Figs 6 and 7). There were no significant differences between participants concerning university, gender, or academic level. However, the percentage of participants from KI that considered ‘communicable’ as very important or greater was significantly higher than the percentage of participants from SSE, χ^2^ (1, N = 34) = 9.41, p < .05 (Fig 8). This indicates that senior researchers in medicine have the opinion that quality research should be communicable to a larger extent than senior researchers in business administration, although the results should be interpreted carefully as we have not set out to examine a representative sample of researchers. The percentage of non-professors among the participants that considered communicable as very important or greater was significantly higher than the percentage of professors among the participants, χ^2^ (1, N = 42) = 4.27, p < .05 (one-sided). Again, our results show that there are differences between subgroups of participants based on their academic discipline and position when it comes to their perceptions of the importance of quality research being communicable.

**Fig 9:**
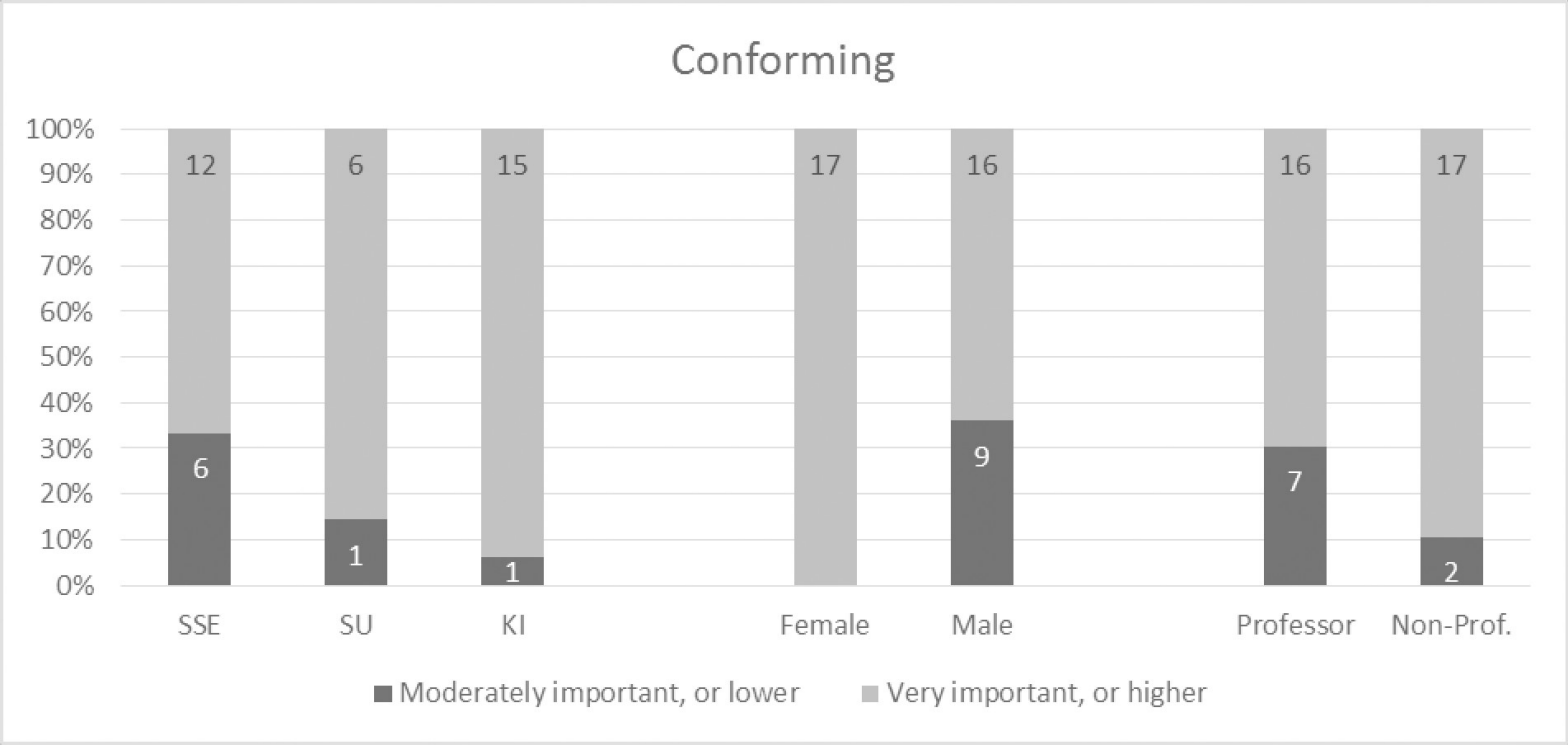
Response rates for conforming between universities, gender and academic level.

Finally, the percentage of female participants that considered conforming as very important or greater was significantly higher than the percentage of male participants, χ^2^ (1, N = 42) = 7.79, p < .01 (Fig 9). Quite interestingly, in the consideration of conforming, there were not any significant differences between the subgroups of participants based on academic discipline or position (cf. communicable).

### Free text comments

The participants were also asked to answer three open, free-text questions to give the further possibility of commenting on the importance of the 32 concepts, or if there were any additional concepts that should be added. These three questions (Q16, Q17, and Q19) and a summary of answers are listed below:

*(Q16) Are any of the 32 concepts that you would consider completely unnecessary*? *If so*, *which and why*?

The majority (39 out of the 42) of the respondents believed that all 32 concepts in the model should be included, but that some could be aggregated into a higher level (similar to ethical). Moreover, three comments indicated that there may actually be too many concepts, for example: *‘Too many concepts*, *perhaps some are more important than others but cannot be ranked in the current format*. *In addition to weights you may consider a ranking procedure*.*’* Further, there were comments on specific items such as: *‘If you do research in sustainable development related topics it is hard to bypass that concept but my suspicion is that it is not otherwise regarded as necessary—along with equal opportunity*.*’* And, *‘many of the concepts are quite obvious for me and relate to research ethics and probably the formula could be built on fewer items*.*’*

From the free-text comments, we would also like to highlight a comment on ‘bypassing’ ethical issues in addition to the sustainable ‘bypassing’ indicated above: *‘Even if it is law*, *it is not really a criterion that is used a lot in the discussion of evaluating dissertations… maybe we should*.*’* As was shown in section 3.4 ‘conforming,’ ethics or morality did not seem to be very important for quality research for a quarter of the participants (23.8% and 26.2% respectively).

*(Q17) Are there any concepts that should be added to the model (please specify in detail below)*?

Only two respondents indicated that new concepts should be added to the model as represented in the following comments: *‘Is the research interesting*? *That would be at the top of my list*.*’* And, *‘your model appears to have been developed for research in the positivist paradigm*. *If you want it to apply to the other research paradigms*, *you will need to add criteria/concepts specific to the other paradigms*. *E*.*g*., *in interpretive research concepts such as trustworthiness*, *engagement*, *educative*, *and ontological authenticity…’*

*(Q19) Other comments on the proposed model*, *on the survey*, *or more general comments*?

The participants’ other comments or general comments were sparse and only eight comments were made. Some of these indicated that the participants thought that the model and/or the survey took too long to grasp, for example: *‘I think 32 variables to check for is too much*!*’* And, *‘the model is too … large*, *several of the aspects are close to each other*.*’* However, none of those participants gave any specific comments in the previous question on which concepts might be unnecessary, therefore making it unclear as to what should be omitted. Moreover, a number of the comments were clearly positive, for example: *‘I have read the paper with interest*.*’* And, *‘I think it could be used as one tool/checklist together with others*.*’*

Finally, some comments provided advice on how the respondent believed that the model could be applied: *‘As many other models for research evaluation*, *it is probably better for groups than individuals*.*’* Further, *‘I think the model might work in particular research traditions*, *not to perform overall assessments of research across disciplines*.*’*

## Discussion

### Main findings

This study presents an interdisciplinary, face validity survey of a quality model of research practice. Only 6 out of 42 participants indicated any of the four main concepts as somewhat important or less, which clearly indicates that the participating senior researchers at these three major Swedish universities see strengths in the model of research quality. Similarly, participants ranked high scores for almost all sub-concepts. The only sub-concepts that might require further discussion are generalizable, original procedure, applicable results, current idea, accessible and searchable, sustainable, and equality. Still, apart for original procedure, about half of the participants indicated these sub-concepts are also of crucial importance or very important.

The comparison between subgroups revealed differences concerning communicable between universities and academic levels and for conforming between genders. The open answers revealed that the majority of participants believe that all concepts in the model are important, but that there might be some that can be aggregated on a higher level. Only two participants indicated new concepts that might be added to the model. Thus, we believe that these findings make an important contribution to the field of research policy and practice. To our knowledge, there have not been any previous evaluation studies of models for research quality in a similar way to the study at hand. The revealed difference concerning conforming between genders is of particular interest in light of recent research on gender effects in research evaluation, where significant gender gaps have been shown [46].

### Rigor vs. relevance

The quality model used the main concept of credible to capture rigor and contributory to capture relevance. At the same time, these two main concepts also capture a broader perspective than rigor and relevance can. Although the model relied on different terminology than previous research [25], participants had no objections to these new terms. Further, we showed that among the participants (senior researchers in various research disciplines), these were both very important and seemed in no way to be ‘either/or’ alternatives. However, we did notice that the concept original procedure was the least important concept of all 32 in the model according to the participants. An original procedure could stand in conflict with credibility if the latter builds on conventional procedures. Our results indicate that credible is perceived as significantly more important, thus supporting previous research that has found rigor to be more important than relevance.

Finally, we showed that communicable and conforming added complexity to previous discussions limited to rigor and relevance, although these were somewhat lower ranked than credible and contributory. There were only differences in perceptions between subgroups of participants about those two additional concepts, perhaps indicating that there is dispersion in how different groups are adjustable to ‘what good research is.’

### Comparison between subgroups

Our study shows tendencies of difference between subgroups of participants based on academic discipline, position, and gender. However, we would like to emphasize that this study did not set out to evaluate a representative sample of senior researchers. Our main aim was to evaluate the face validity of a previously published quality model of research practice. As we have relied on convenience sampling, we have not attempted to generalize the findings of differences between the subgroups in our limited sample of 42 participants. Nevertheless, our results indicate that there could be differences between subgroups based on their academic achievements, environment, and other dimensions which lead to different perceptions of research quality. Our finding of difference between the genders of quality perceptions in research could be a potential explanation of the gender gap in research evaluations as discussed by Jappelli et al. [46]. We therefore propose to investigate those differences further in future research.

### Free text comments

It is interesting to note that specific sub-concepts such as sustainable or ethical–concepts that in most countries (and especially in Sweden) are today seen as outmost important–seem to trigger reflections on ‘bypassing’ (see quote in section 3.7, free text comments). We have not been able to find discussions in the scientific literature regarding these somewhat challenging thoughts, therefore we see this as a need for investigation in future research.

### Example use of the weighted model

One specific way of using the proposed model is to first discuss what criteria should be used in the evaluation of research. Then, as a second step, different weights can be allocated to the different dimensions used. In a specific situation where the model should be used to evaluate research, a simplified version of the process could look like the following:

1. The management team of a university selects members of an evaluation committee with the task of evaluating certain research projects or similar.
2. Within the committee, the members take their starting-point in the research quality model and discuss if there are aspects of it that they feel an urgent need to change; e.g., if there are concepts that should be added given the specific context. Most likely, this would rarely be the case, given the deliberate general nature of the model.
3. When the quality model is agreed upon within the evaluation committee, there is a discussion about the weighting of the different concepts. There may be overall aims with the research at the university, or there may be instructions to the committee regarding certain aspects of research that are especially important or not. Based on the instructions, the members of the committee then decide on the different weights attributed to the model’s concepts. The default is that all concepts are of equal weight. If one concept is perceived to be of greater importance in the evaluation of the research projects, it can then be allocated a higher weight. If another concept is perceived to be of less importance, it could consequently be allocated a lower weight.
4. The members of the committee can start evaluating the different projects according to all the included concepts. By operationalizing the different concepts, the collection and analysis of the different members’ evaluations will become easier. Furthermore, the members of the committee can, in their discussions about how to evaluate different specific research projects, take a starting-point in different dimensions which they have already agreed upon using when evaluating the project at hand.

### Strengths and limitations

The face validity evaluation process of the quality model presented in this paper has a number of strengths. First, the face validity process was carried out by a number of senior researchers in various fields. This strategic sample gives a seniority which is in line with the face validity intention. Second, to our knowledge, this is the first time a similar validity evaluation has been conducted for a research quality evaluation model. Third, the questionnaire used in the survey had a high response rate from senior researchers in a wide range of disciplines.

Nevertheless, there are also some limitations. First, the number of participants in the survey was rather limited, although this was deliberate. Specifically, the study aimed to get input from a reasonable number of researchers representing various subject areas. An implication of this is that we were not only interested in their answers concerning the 32 different concepts, but also their views of the model. In light of this, we believe that a limited sample size, such as in this survey, makes sense. Second, we realize that the questionnaire was a bit challenging to answer, given all the different concepts and descriptions. Thus, its complexity was yet another argument to keep the number of respondents limited.

### Implications for policymakers, researchers, and future research

The quality model first presented in Mårtensson et al. [10] has now been evaluated regarding its face validity, and no major changes need to be made. An implication from this is that policymakers and other stakeholders now have a ready-to-use quality model to evaluate research, whether this is in an academic or practical context, in terms of, for example, R&D projects. The model can be used at different stages of a research process–before, during, and after–and by different stakeholders for different purposes. Decision-makers at foundations allocating research funding can apply the quality model in order to structure the evaluation of research projects ‘before’ the research has been conducted. Researchers can apply the model in their ongoing projects to facilitate a multi-perspective view, and what aspects they pay attention to, or not, ‘during’ research. Managers can apply the model when monitoring R&D projects. Further, evaluators at foundations can apply the model when evaluating research projects that are reported back to foundations ‘after’ the research has been conducted.

An alternative way of validating the quality model is to conduct an inductive interview study among, for example, decision-makers at research foundations. Through such a procedure, their current practices can be compared to the quality model, and thereby the understanding of the evaluation process can be improved. Another path forward for this research is to conduct a large-scale test of the model, to make more detailed comparisons between different subject areas as well as different geographical regions and so forth. A third path for future research is to apply the quality model in a detailed case study, and through that process collect data from the different stakeholders involved in order to learn about any differences.

## Concluding remarks

The research quality model based on four basic concepts and 28 sub-concepts has–overall–been found valid, as an important contribution for describing the quality of research practice, by 42 senior researchers from three major Swedish universities. The majority of the respondents believed that all concepts in the model are important, and did not indicate the need for further development or the addition of new concepts. Thus, the face validity of the quality model seems to be high. However, some of the sub-concepts were indicated as slightly less important. Moreover, there were significant differences concerning communicable between disciplines (universities) and academic levels, and for conforming between genders.

Our study illustrates that the research quality model opens up the opportunity to move from working with two traditional core concepts (rigor and relevance), akin to merely ‘two leaves in the concept tree’ per se, to instead working with 32 concepts and the whole tree. Future research regarding a more systematic approach to research quality improvement therefore may be met by the nuances offered within such a comprehensive concept hierarchy.

## Supporting information

S1 Appendix The questionnaire. (PDF)

S1 Table Detailed responses to the questionnaire, percentages of responses per concepts. (PDF)
