# Supplementary material for: Quality of Research Practice – An interdisciplinary face validity evaluation of a quality model
Source: PLoS One. 2019 Feb 1;14(2):e0211636. doi: 10.1371/journal.pone.0211636 (PMC6358077; doi:10.1371/journal.pone.0211636)
Supplement: S1 Appendix — (PDF) [file pone.0211636.s001.pdf]

S1 Appendix: The Questionnaire

Background information

Q1 Your research field?

Q2 Current institution/university?

Q3 Current department?

Q4 Age?

Q5 Gender?

Q6 Highest academic degree attained?

Q7 Institution/University granting the highest degree and year:

Q8 The scientific area your highest academic degree is in (e.g., history, medicine, management etc.):

Q9 Current position:

Q10 Possible current academic leadership position? (dean, director of studies, head of department, etc.)

Q11 Do you have tenure/a permanent position?

Q12 In which country have you mainly been active as a researcher?

13 In which countries have you also been doing research actively for at least 3 months?

Q14 Approximately, how many PhD or licentiate dissertations have you been involved in as a member of the grading committee and/or as an opponent?

The model for research evaluation is based on 32 concepts (please refer to the article for more details) that each can be more or less applicable to a certain research field. We would like you, as an expert in your field, to indicate the extent to which these concepts are important when you evaluate research in your field on the scale 'Not at all important', 'Somewhat important', 'Moderately important', 'Very important', 'Of crucial importance'.

Q15 To evaluate the quality of a dissertation in your field, how important are the following concepts?

	Not at all important (1)	Somewhat important (2)	Moderately important (3)	Very important (4)	Of crucial importance (5)
1. Credible (The research is Coherent, Consistent, Rigorous, and Transparent)	❍	❍	❍	❍	❍
2. Rigorous (The research is Contextual, Internally Valid, and Reliable)	❍	❍	❍	❍	❍

3. Consistent (New Knowledge is logically linked to Existing Knowledge and is in accordance with the Scientific Method and Question at Hand).	❍	❍	❍	❍	❍
4. Coherent (Adequate consideration is given to Existing Knowledge in the chosen Context).	❍	❍	❍	❍	❍
5. Transparent (Relevant New Knowledge in the reporting of research results is included and the process is described in relation to the Question at Hand, Scientific Method, and Existing Knowledge).	❍	❍	❍	❍	❍
6. Internally Valid (A correct Scientific Method (incl. research design) is used in relation to the Question at Hand and Context, and New Knowledge is Provable).	❍	❍	❍	❍	❍
7. Reliable (The chosen Scientific Method is appropriate for the present Question at Hand and Context, and is documented in a Described Procedure that others could use to reach a similar result in the same Context).	❍	❍	❍	❍	❍
8. Contextual (Existing Knowledge that is relevant for the Context is used, and is presented according to Rules for Description).	❍	❍	❍	❍	❍
9. Contributory (The research is Original, Relevant, and Generalizable).	❍	❍	❍	❍	❍
10. Original (The research has an Original Idea, uses an Original Procedure and produces an Original Result).	❍	❍	❍	❍	❍
11. Relevant (The research has a Relevant Research Idea, Applicable Result and Current Idea).	❍	❍	❍	❍	❍
12. Generalizable (New Knowledge is practically or theoretically useful in Contexts other than the one studied).	❍	❍	❍	❍	❍
13. Original in its Idea (The Question at Hand has not been asked before in the current Context or is interpreted in a novel way).	❍	❍	❍	❍	❍
14. Original in its Procedure (The described Procedure is original in relation to the Question at Hand).	❍	❍	❍	❍	❍
15. Original in its Result (New Knowledge is Provable in relation to Existing Knowledge).	❍	❍	❍	❍	❍
16. Relevant Research Idea (The question at Hand is relevant for the current Target Group).	❍	❍	❍	❍	❍
17. Applicable in its Result (New knowledge is Beneficial for the current Target Group).	❍	❍	❍	❍	❍
18. Current in its Idea (The Question at Hand is in accordance with the current Context).	❍	❍	❍	❍	❍
19. Communicable (The research is Consumable, Accessible, and Searchable).	❍	❍	❍	❍	❍
20. Consumable (The research is Structured, Understandable, and Readable).	❍	❍	❍	❍	❍
21. Accessible (New Knowledge is easily	❍	❍	❍	❍	❍

available to the Target Group).					
22. Searchable (The documented New Knowledge is structured according to the Rules for Description and easily found by the Target Group).	❍	❍	❍	❍	❍
23. Structured (The Research documentation follows the Rules for Description).	❍	❍	❍	❍	❍
24. Understandable (The language in the Research documentation is understandable for the Target Group).	❍	❍	❍	❍	❍
25. Readable (A Correct language is used in the Research documentation for the Target Group).	❍	❍	❍	❍	❍
26. Conforming (The research is Aligned with Regulations, Ethical, and Sustainable).	❍	❍	❍	❍	❍
27. Aligned with Regulations (The Research complies with currently applicable legal aspects of the System of Rules).	❍	❍	❍	❍	❍
28. Ethical (The Research is Morally Justifiable, Open, and supports Equal Opportunities).	❍	❍	❍	❍	❍
29. Sustainable (The Research complies with sustainable development aspects as described in the System of Rules).	❍	❍	❍	❍	❍
30. Morally Justifiable (The Research complies with currently applicable ethical standards as described in the System of Rules).	❍	❍	❍	❍	❍
31. Open (The Research demonstrates Transparency with currently applicable ethical standards as described in the System of Rules).	❍	❍	❍	❍	❍
32. Considering Equal Opportunities (The Research is consistent with equal treatment according to the System of Rules)	❍	❍	❍	❍	❍

Q16 Are any of the 32 concepts above completely unnecessary? If so, which and why?

Q17 Are there any concepts that should be added to the model (please specify in detail below?)

General Comments

Q18 In general, for what purpose(s) do you think this type of model could be useful? (You may tick more than one).

- ❑ To evaluate applications for research funding
- ❑ To evaluate if dissertations should pass
- ❑ To review scientific manuscripts
- ❑ To evaluate research of a university
- ❑ To compare research quality within a university
- ❑ Other ____________________

Q19 Other comments on the proposed model, on the survey, or more general comments.
