# Supplementary material for: Quality of Research Practice – An interdisciplinary face validity evaluation of a quality model
Source: PLoS One. 2019 Feb 1;14(2):e0211636. doi: 10.1371/journal.pone.0211636 (PMC6358077; doi:10.1371/journal.pone.0211636)
Supplement: S1 Table — (PDF) [file pone.0211636.s002.pdf]

S1 Table: Detailed responses to the questionnaire, percentages of responses per concepts

Rank	*Credible*	*Rigorous*	*Consistent*	*Coherent*	*Transparent*	*Internally valid*	*Reliable*	*Contextual*
1								
2						2.4	7.1	
3	4.8	7.1	9.5	21.4	9.5	19.0	7.1	21.4
4	26.2	38.1	52.4	38.1	52.4	28.6	35.7	42.9
5	69.0	54.8	38.1	40.5	38.1	50.0	50.0	35.7

Rank	*Contributory*	*Original*	*Relevant*	*Generalizable*	*Original (idea)*	*Original (procedure)*	*Original (results)*	*Relevant (idea)*	*Applicable (results)*	*Current (idea)*
1						2.4		2.4	4.8	4.8
2	2.4	4.8	4.8	4.8	9.5	16.7	4.8	2.4	16.7	11.9
3	9.5	33.3	11.9	42.9	26.2	45.2	33.3	21.4	21.4	33.3
4	52.4	40.5	50.0	33.3	40.5	33.3	50.0	38.1	38.1	33.3
5	35.7	21.4	33.3	19.0	23.8	2.4	11.9	35.7	19.0	16.7

Rank	*Communicable*	*Consumable*	*Accessible*	*Searchable*	*Structured*	*Understandable*	*Readable*
1			2.4	4.8			
2	9.5	2.4	11.9	7.1	4.8	2.4	4.9
3	28.6	12.2	35.7	38.1	16.7	29.3	24.4
4	35.7	53.7	28.6	31.0	47.6	34.1	34.1
5	26.2	31.7	21.4	19.0	31.0	34.1	36.6

Rank	*Conforming*	*Compliant*	*Ethical*	*Sustainable*	*Moral*	*Open*	*Equality*
1	2.4	2.4	4.8	7.1	2.4		4.8
2	4.8		2.4	9.5	4.8	9.5	11.9
3	14.3	23.8	16.7	33.3	19.0	14.3	26.2
4	26.2	33.3	23.8	28.6	21.4	38.1	33.3
5	52.4	40.5	52.4	21.4	52.4	38.1	23.8

Note: Rank was measured on (1) not important at all, (2) partly important, (3) important, (4) very important, and (5) of crucial important
